# Detecting the impacts of humidity, rainfall, temperature, and season on chikungunya, dengue and Zika viruses in *Aedes albopictus* mosquitoes from selected sites in Cebu city, Philippines

**DOI:** 10.1186/s12985-024-02310-4

**Published:** 2024-02-15

**Authors:** Frances Edillo, Rhoniel Ryan Ymbong, Anthoddiemn Olin Navarro, Maureen Mathilde Cabahug, Kristilynn Saavedra

**Affiliations:** 1 Mosquito Research Laboratory, Department of Biology, University of San Carlos– Talamban Campus, 6000 Cebu city, Philippines; 2 Department of Science and Technology, Science Education Institute, Taguig City, Metro Manila 1631 Philippines

**Keywords:** Chikungunya, Dengue, Zika, Asian tiger mosquitoes, Arboviral diseases

## Abstract

**Background:**

*Aedes albopictus* is the secondary vector for dengue virus (DENV) in the Philippines, and also harbors chikungunya (CHIKV) and Zika (ZIKV) viruses. This study aimed to determine the minimum infection rates (MIRs) of CHIKV, DENV serotypes, and ZIKV in *Ae. albopictus* collected from selected two-site categories by altitude (highland [H] and lowland [L] sites) in Cebu city, Philippines during the wet (WS) and dry seasons (DS) of 2021–2022, and to explore the relationships between these arboviral MIRs and the local weather.

**Methods:**

The viral RNA extracts in pooled and reared adult *Ae. albopictus* collected during the DS and WS from two-site categories were subjected to RT-PCR to amplify and detect gene loci specific for CHIKV, DENV-1 to DENV-4, and ZIKV and analyzed with the weather data.

**Results:**

The range of CHIKV MIRs was higher in the WS (13.61–107.38 infected individuals per 1,000 mosquitoes) than in the DS (13.22–44.12), but was similar between the two-site categories. Rainfall (RF) influenced the CHIKV MIR. The MIR ranges of both DENV-2 (WS: H = 0, L = 0; DS: H = 0–5.92; L = 0–2.6) and DENV-4 (WS: H = 0, L = 0–2.90; DS: H = 2.96–6.13, L = 0–15.63) differed by season but not between the two-site categories. Relative humidity (RH), RF, and temperature did not influence DENVs’ MIRs. The MIR range of ZIKV was similar in both seasons (WS: 11.36–40.27; DS: 0–46.15) and two-site categories (H = 0–90.91, L = 0–55.56). RH and temperature influenced ZIKV MIR.

**Conclusions:**

RF influenced CHIKV MIR in *Ae. albopictus*, whereas RH and temperature influenced that of ZIKV. Season influenced the MIRs of CHIKV and DENVs but not in ZIKV. *Ae. albopictus* were co-infected with CHIKV, DENVs, and ZIKV in both highland and lowland sites in Cebu city. Recommendations include all-year-round implementation of the Philippine Department of Health’s * 4S* enhanced strategy and installation of water pipelines in rural highlands for vector and disease control. Our findings are relevant to protect public health in the tropics in this climate change.

**Supplementary Information:**

The online version contains supplementary material available at 10.1186/s12985-024-02310-4.

## Background

While *Aedes aegypti* (Linnaeus) is the main vector of arboviruses such as chikungunya (CHIKV), dengue (DENV), and Zika (ZIKV), *Aedes albopictus* (Skuse), the Asian tiger mosquito, plays also a role in its prevalent arboviral transmission to humans [1–4] throughout the tropics and subtropics [5–8]. Dengue fever is the most common global arboviral infection; 3.6 billion people reside in dengue-endemic areas [9]. Chikungunya reached its global significance in the 21st century [10], whereas Zika caused 7.6 million cases globally in 2016 [11]. In the Philippines, chikungunya infections (600 cases; 0 death) were 545% higher from January 1 to December 31, 2022 than in the same period in 2021. Of which, 127 cases occurred in Central Visayas, where Cebu city belongs [12]. The Philippine dengue surveillance (61,170 cases; 216 deaths; case fatality rate [CFR] = 0.4%) ranked second and first in the number of cases and deaths, respectively, in the Western Pacific Region in 2021 [13]. Of which, 2,604 cases and 17 deaths (CFR = 0.7%) occurred in Central Visayas during the same period of 2021 [12]. The same trend in 2022 was reported in the Philippines (205, 679 cases; 672 deaths; CFR = 0.3%) [14]; of which 17,894 dengue cases (104 deaths; CFR = 0.6%) were reported in Central Visayas [12]. Meanwhile, the first 17 Zika cases were reported in the Philippines in 2016 without travel history from an affected country a month prior to their onset of illness [15]. No Zika case was reported since 2016, but because *Ae. aegypti* and *Ae. albopictus* are found in the country, CHIKV and ZIKV might have been circulating around.

Most studies revealed that temperatures influence large epidemics of mosquito-borne arboviral diseases [16–18]. In Latin America, temperature and its range, rainfall (RF), and population size are the important predictors of Zika transmission [19]. In Brazil, RF predicts ZIKV and CHIKV with a lead time of three weeks each time [20]. In Mexico, the dynamics of dengue, chikungunya, and Zika are strongly associated with seasonal climatological variability and transmission potential of these arboviral pathogens [21]. In Asia, only a few studies explored the relationship between arboviral mosquito-borne diseases and the weather. In India, Shil et al. [22] observed a strong positive association between the occurrence of CHIKV cases and RF variations. In Japan, Furuya’s model [23] predicts that if the daily mean temperature rises from 28 to 30 °C, the median arboviral reproduction number (R_0_) increases by 18% for dengue, 4.3% for chikungunya, and 11.1% for Zika. However, most studies monitored only CHIKV and ZIKV in *Aedes* such as in Asia-Pacific [24], Hindu-Kush Himalayan region [25], Singapore [26], Sri Lanka [27], and Thailand [7]. In the Philippines, only Balingit et al. [28] and Edillo et al. [29] reported DENVs in *Ae. aegypti,* the primary dengue mosquito vector, in Tarlac and Cebu cities, Philippines, respectively. However, no study, to our knowledge, has determined the influence of the weather to the infections of CHIKV, DENVs, and ZIKV in the Philippine *Ae. albopictus*, the secondary dengue mosquito vector, in this current climate change.

The National Dengue Prevention and Control Program of the Philippine Department of Health (DOH) [30] includes the following components: (1) surveillance through the Philippine Integrated Disease Surveillance and Response, (2) case management and diagnosis, (3) integrated vector management, (4) outbreak response, (5) health promotion and advocacy, and (6) research. The enhanced *4 S* strategy [12] has been the main focus for dengue prevention and control, where “*4S*” stands for the following: (1) seek and destroy mosquito-breeding sites, (2) seek early consultation if one develops dengue-associated symptoms, (3) employ self-protection measures such as wearing long pants and long-sleeved shirts, and (4) say “no” to indiscriminate fogging, and implement fogging only during outbreaks in hotspots. Climate change affects the distribution of *Ae. aegypti* and *Ae. albopictus*, which allows them to spread new pathogens to initially-unaffected populations in the mountains [31]. The latter calls for urgent actions on vector control strategies [32], including source reduction of vectors, to manage the spread of these arboviral diseases, and to protect public health [33–35] considering the lack of effective vaccines available.

We hypothesized that weather conditions might influence CHIKV, DENVs, and ZIKV in *Ae. albopictus* from selected highland and lowland sites in Cebu city, Philippines during the wet (WS) and dry seasons (DS). Thus, this study aimed: (1) to determine the presence and calculate the minimum infection rates (MIRs) of CHIKV, DENV-1 to DENV-4, and ZIKV in *Ae. albopictus* collected from selected two-site categories by altitude (highland and lowland sites) in Cebu city during the 2021–2022 WS and DS, and (2) to explore the relationships among the arboviral MIRs with the local weather in the study sites. *Ae. albopictus* was chosen in this study for the following reasons: (1) there has been no study in the Philippines, to our knowledge, that reported the impacts of weather, season, and altitude to the arboviral infections in this mosquito species. (2) Arboviral monitoring in *Ae. albopictus* is a relevant strategy for tracking their activities and for implementing direct control actions [28, 30, 36] against associated diseases and vectors as influenced by the weather. However, the local Philippine DOH focuses in monitoring the number of suspected arboviral mosquito-borne diseases in humans and not in mosquito vectors, and community participation of the enhanced * 4S* strategy. (3) Also, *Ae. albopictus* is a good sentinel species in DENV prevalence during the inter-epidemic period [37] in this climate change.

## Methods

### Study sites

**Fig. 1:**
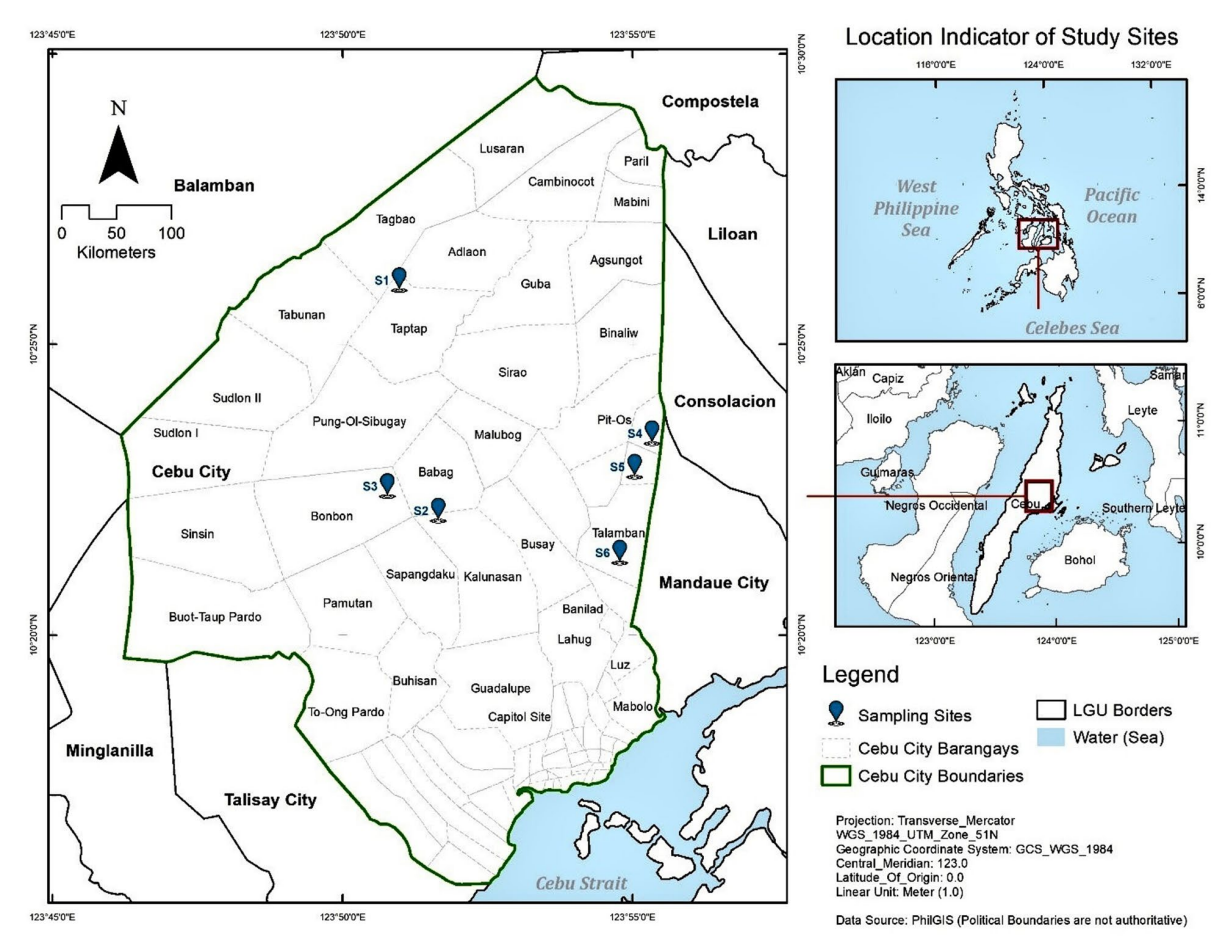
Map of the six highland (S1: Taptap, S2: Babag 1 and S3: Babag 2) and lowland (S4: Pit-os, S5: Bacayan, S6: Talamban) sites in Cebu city, Cebu, Philippines

The study sites in Cebu city, Philippines were selected *barangays* (i.e., smallest government units) based on: (1) altitude (highland vs. lowland), (2) presence of local dengue cases, (3) vegetation cover, and (4) in-between site distance of more than 500 m (Table 1; Fig. 1). Highland sites had elevations between 400 and 700 m above sea level (m ASL); lowland sites, between 60 and 80 m ASL.

**Table 1:** Coordinates and elevation of the six highland and lowland sites in Cebu city

Study Sites (Code number)	Coordinates	Elevation (m ASL)
Highlands		
Taptap (S1)	N 10.42619°; E 123.8442°	719
Babag– 1 (S2)	N 10.36876°; E 123.8601°	617
Babag– 2 (S3)	N 10.37282°; E 123.8459°	405
Lowlands		
Bacayan (S4)	N 10.38690°; E 123.91910°	83
Pit-os (S5)	N 10.40160°; E 123.91910°	83
Talamban (S6)	N 10.35373°; E 123.91264°	65

### Mosquito collections

*Aedes* eggs and sub-adults were collected bimonthly during the DS (March– May 2021 and 2022) and WS (June– November 2021; February and June 2022) using the modified ovicidal/larvicidal (O/L) traps or ovitraps (Department of Science and Technology, Manila). Briefly, the ovitrap consisted of a black plastic container, filled halfway with water, and a filter paper-wrapped wooden panel. The original O/L pellets were discarded to allow the oviposited *Aedes* eggs and sub-adults to survive [34, 35].

Each highland and lowland site had 10 modified O/L traps, placed in locations with thick vegetation cover. The traps were left for 10–15 days. The filter papers with oviposited *Aedes* eggs (referred to as “egg papers” henceforth) were collected, dried, placed in a clean and dry container and stored in a dark place. Sub-adults were collected from the traps or other water-filled containers within the vicinity by scooping with nets or aspirating with Pasteur pipettes.

### Rearing of collected *Aedes* eggs and sub-adults

Field-collected *Aedes* egg papers and sub-adults were transported to the University of San Carlos - Mosquito Research Laboratory (USC-MRL), Biology Department, Talamban campus, Cebu city, Philippines. *Aedes* egg hatching was performed by submerging the egg papers in 10% ascorbic acid solution [38]. *Aedes* larvae and pupae were transferred to a clean plastic container filled halfway with distilled water (DW) and covered with a fine-mesh cloth. They were fed daily with a dash (~ 0.02 g) of fish food (Sakura; All Aquariums Co., Ltd., Thailand). The DW in the larval plastic container was changed regularly to prevent the formation of bacterial scum until adult emergence. Adult *Ae. albopictus* samples were identified [39] and transferred to a separate container. They were fed with 10% sucrose solution for five days, individually transferred to a microcentrifuge tube, and stored in a -80 °C ultralow freezer until further processing.

### Sample size

The sample size was calculated to consider the difference between binomial proportions (i.e., detection or non-detection) [40] of CHIKV, DENVs, and ZIKV in *Ae. albopictus* collected from highland and lowland sites of Cebu city during the DS and WS. Neyman allocation was used as an adjunct for generating a sample size for each site (i.e., apportioned from computed total sample size) with an allocated fixed budget [29]. At the significance level (α) of 0.05, 80% power (β = 0.2), probabilities p1 = 0.10 and p2 = 0.30 (based from the detection of Guedes et al. [41] and Medeiros et al. [36], respectively) and 20% attrition rate, four pools (i.e., a pool ≤ 30 adults) of *Ae. albopictus* collected from each site monthly for DS and three pools collected from each site monthly for WS were considered sufficient to warrant rejecting the hypothesis of no difference. A total of 288 pools of *Ae. albopictus* were prepared for both the DS (*n* = 144 pools) and the WS (*n* = 144 pools).

### RNA extraction

RNA extraction of adult *Ae. albopictus* was performed using the RNeasy mini kit (Qiagen, Germany) following the manufacturer’s protocol. The quality of RNA extraction was determined by gel electrophoresis. RNA extracts of *Ae. albopictus* were expected to produce 18s and 28s  ribosomal RNA bands at 1,000 and 1,500 base pairs (bp), respectively [42]. All extracted sample pools were composed of either male or female, or mixed adults.

### Detection of CHIKV, DENV, and ZIKV

#### Reverse transcription-polymerase chain reaction (RT-PCR)

One-step RT-PCR was performed to detect these arboviruses in pooled *Ae. albopictus* samples using Qiagen kit (Germany; Cat No. 210212). Primer sequences are listed in Additional file 1: Table S1.

#### CHIKV PCR

CHIKV PCR reaction was composed of 2.5 µL 5x RT-PCR buffer, 0.5 µL deoxynucleotide triphosphate (dNTP) mix (10 µM), 0.5 µL enzyme mix, 0.25 µL 6 K/E1 OA (10 µM) and 1 µL 6 K/E1 OB (10 µM) primers, 1.25 µL RNA template, and 6.5 µL RNAse-free water (RFW). The RT-PCR profile was set at 50˚C RT for 30 min, 95˚C incubation for 10 min, 40 cycles of 95˚C denaturation for 30 s, 53˚C annealing for 30 s, 72˚C extension for 30 s, and 72˚C final extension for 10 min.

#### Nested PCR of CHIKV

CHIKV nested PCR was done to further amplify the inner region of 6 K/E1 gene. A nested PCR concoction (10 µL) was prepared: 5 µL 2x PCR mastermix, 0.5 µL of both inner 6 K/E1 IA and 6 K/E1 IB primers, 1 µL cDNA template and 3 µL RFW.The PCR profile was set at 95˚C incubation for 10 min, 35 cycles of 95˚C denaturation for 30 s, 55˚C annealing for 30 s, 72˚C extension for 30 s, and 72˚C final extension for 10 min. CHIKV outer and inner primers used in nested RT-PCR were based on Laras et al. [43].

#### DENV PCR

The DENV PCR reaction contained 2.5 µL 5x buffer with 1.5 mM MgCl2, 0.5 µL dNTPs (10 Mm), 0.5 µL RT- PCR enzyme mix, 0.75 µL D1 (10 µM) and 0.75 µL D2 (10 µM) primers, 2 µL RNA sample, and 5.5 µL RFW. The PCR profile was set at 50 °C RT for 30 min, 95 °C incubation for 15 min; 35 cycles of 94 °C denaturation for 30 s, 55 °C annealing for 1 min, 72 °C extension for 1 min, and final extension at 72 °C for 10 min. After RT-PCR, a second PCR was done using similar PCR reaction and conditions to visualize distinct bands in gel electrophoresis.

#### Serotyping of DENVs

Products from the DENV RT-PCR were used for DENV serotyping using HotStarTaq master mix kit (Qiagen, Germany; Cat. No. 203446) with PCR profile of 95 °C incubation for 15 min, 25 cycles of 94 °C denaturation for 30 s, 55 °C annealing for 1 min, 72 °C extension for 1 min, and final extension at 72 °C for 10 min.The DENV serotyping PCR reaction was made up of 12.5 µL 2x HotStarTaq master mix, 1 µL 25 mM MgCl2, 0.5 µL dNTPs (10 Mm), 1.5 µL cDNA template, 6.375 µL NFW, and 0.625 µL of each of the following primers (10 µM) such as D1, D2, TS1, TS2, TS3, and TS4. DENV PCR and serotyping were based on Lanciotti et al. [44].

#### ZIKV PCR

ZIKV PCR reaction was made of 2.5 µL 5x RT-PCR buffer, 0.5 µL dNTP mix (10 µM), 0.5 µL enzyme mix, 0.625 µL ZIKV (10 µM) forward and reverse primers, 2 µL RNA template, and 5.75 µL RFW. The RT-PCR profile was set at 50˚C RT for 30 min, 95˚C incubation for 10 min, 40 cycles of 95˚C denaturation for 30 s, 57˚C annealing for 30 s, 72˚C elongation for 30 s, and 72˚C final elongation for 10 min. The protocol was based on Balm et al. [45] with modifications.

### PCR product purification and sequencing of positive controls

Positive controls for CHIKV and ZIKV amplicons were isolated from local *Ae. albopictus*, whereas those for DENV-1 to DENV-4 were isolated from *Ae. aegypti* or *Ae. albopictus* (Table 2). They were subjected to the aforementioned RNA extraction and RT-PCR protocols. For confirmation of arboviral amplicons, they were electrophoresed on 1% agarose gel at 200 VDC for 20 min.

**Table 2:** Positive control profiles of the three arboviruses isolated from the local *Aedes* species

Arbovirus	Expected band size (bp)	Aedes species	Site of Origin	Date of Collection
CHIKV	200 (49)	*Ae. albopictus*	Talamban (S6)	August 2021
DENV (consensus)	511 (50)	*Ae. aegypti*	Liloan, Cebu	August 2021
DENV-1	482 (50)	*Ae. aegypti*	Liloan, Cebu	August 2021
DENV-2	119 (50)	*Ae. albopictus*	Talamban (S6)	November 2021
DENV-3	290 (50)	*Ae. aegypti*	Liloan, Cebu	October 2021
DENV-4	392 (50)	*Ae. aegypti*	Liloan, Cebu	November 2021
ZIKV	192 (51)	*Ae. albopictus*	Taptap (S1)	August 2021

Non-specific amplifications were observed among the PCR products of positive controls, thus, gel excision and extraction of the bands corresponding to expected band size of each arbovirus (Table 2) were done using QIAquick PCR and gel cleanup kit (Qiagen, Germany; Cat. No. 28704) following the manufacturer’s protocol. Another PCR run was done to further amplify the desired DNA fragment using Type-it microsatellite PCR kit (Qiagen, Germany). A PCR reaction (20 µL) was made of 10 µL 2x PCR mastermix, 1 µL (10 µM) each of the forward and reverse primers, 4 µL DNA template, and 4 µL RFW. The PCR profile for CHIKV positive control was set at 95˚C incubation for 10 min, 35 cycles of 95˚C denaturation for 30 s, 53˚C annealing for 30 s, 72˚C extension for 30 s, and 72˚C final extension for 10 min. For DENVs and ZIKV positive controls, the PCR profile was similar except annealing temperatures (i.e., 55˚C for DENV and 57˚C for ZIKV).

Purified PCR products for CHIKV, DENV-1 to DENV-4, and ZIKV positive controls were brought to the Philippine Genome Center, University of the Philippines - Diliman, Quezon city for capillary sequencing. Their results were processed using DNA Baser Assembler v4, and were trimmed and cleaned using BioEdit v7.2 [46]. The final sequences were subjected to the Basic Local Alignment Search Tool (BLAST) of the National Center for Biotechnology Information (NCBI) [47] for identity verification.

### Weather data

The mean monthly relative humidity (RH), monthly total rainfall (RF), and mean monthly temperature nearest (11.8– 32.4 km) to the study sites throughout the study period were obtained from the local satellite office of the Philippine Atmospheric Geophysical and Astronomical Services Administration (PAG-ASA), Cebu city.

### Data and statistical analyses

The monthly MIRs of CHIKV, DENV-1 to DENV-4, and ZIKV were computed for each site by dividing the total number of positive pools by the total number of individual mosquito samples, multiplied by 1,000 [48]. Because of non-normality of data distributions, the dependent variables (arboviral MIRs) and the independent variables (season, altitude, and weather data) were subjected to Kruskal-Wallis (K-W) statistical tests with arbitrarily categorized ranks for weather data (Table 3) by using SPSS Advanced Professional (v28). Spearman’s rank coefficient of correlations were performed between the MIRs of CHIKV and ZIKV, and the weather with significant K-W test results to assess the correlation. Pearson’s product-moment correlation tests were performed between the MIRs of DENVs and the dengue cases. Two-tailed Student’s t-tests were determined for the statistical differences between seasonal weather data.

**Table 3:** Categorized ranks for weather data (mean monthly relative humidity [RH], monthly total rainfall [RF] and mean monthly temperature) utilized in Kruskal-Wallis statistical tests

Weather Condition	Low	Moderate	High
RH	74–76%	77–78%	79–80%
RF	< 120 mm	120–150 mm	> 150 mm
Temperature	27 °C	28 °C	29 °C

## Results

### Isolation of CHIKV, DENVs, and ZIKV in *Ae. albopictus* as positive controls

The positive controls isolated from the local *Ae. albopictus* formed the expected gel bands consistently at 200 bp for CHIKV [43], 511 bp for DENV [44], and192 bp for ZIKV [45] (Table 2). The NCBI BLAST results of amplicon nucleotide sequences of these bands yielded 100% identity matches. Likewise, the positive controls with their expected gel bands for DENV-1 (482 bp), DENV-3 (290 bp), and DENV-4 (392 bp) isolated from the local *Ae. aegypti*, and for DENV-2 (119 bp) isolated from the local *Ae. albopictus* were formed consistent with Lanciotti et al. [44].

### MIRs of CHIKV in *Ae. albopictus* by season and altitude

Table 4 (Additional file 2: Dataset 1) shows that during the DS, the highest MIR of CHIKV (47.45 per 1,000 mosquitoes in the wild; from here onward MIR refers to an estimate for every 1,000 *Ae. albopictus* in the wild) was recorded in the lowland Talamban site. The lowland Bacayan had the lowest MIR (15.63) in the DS and in the entire sampling period; likewise it had also the lowest MIR (40.00) in the WS. The lowland Pit-os had the highest MIR (57.97) of CHIKV during the WS and in the entire sampling period.

**Table 4:** MIRs of CHIKV, DENV and ZIKV with number of positive pools in *Ae. albopictus* by season (dry and wet) and two-site categories by altitude (highland and lowland) in Cebu city, Philippines

Season and Sites	n^a^	MIRs (w/ number of positive pools^b^)					
		CHIKV	DENV-1	DENV-2	DENV-3	DENV-4	ZIKV
Dry Season							
Highland							
Taptap (S1)	358	33.52 (12)	0.00 (0)	0.00 (0)	2.79 (1)	5.59 (2)	27.93 (10)
Babag 1 (S2)	326	18.40 (6)	3.07 (1)	3.07 (1)	6.13 (2)	6.13 (2)	36.82 (12)
Babag 2 (S3)	338	32.54 (11)	2.96 (1)	5.92 (2)	8.88 (3)	2.96 (1)	17.75 (6)
Lowland							
Bacayan (S4)	384	15.63 (6)	7.81 (3)	2.60 (1)	13.02 (5)	15.63 (6)	31.25 (12)
Pit-os (S5)	319	34.48 (11)	0.00 (0)	0.00 (0)	9.40 (3)	3.13 (1)	18.81 (6)
Talamban (S6)	274	47.45 (13)	7.30 (2)	0.00 (0)	3.65 (1)	0.00 (0)	18.25 (5)
Wet Season							
Highland							
Taptap (S1)	313	51.12 (16)	0.00 (0)	0.00 (0)	12.78 (4)	0.00 (0)	31.95 (10)
Babag 1 (S2)	312	48.08 (15)	3.21 (1)	0.00 (0)	0.00 (0)	0.00 (0)	32.05 (10)
Babag 2 (S3)	335	47.76 (16)	2.99 (1)	0.00 (0)	0.00 (0)	0.00 (0)	17.91 (6)
Lowland							
Bacayan (S4)	475	40.00 (19)	4.21 (2)	0.00 (0)	4.21 (2)	0.00 (0)	27.37 (13)
Pit-os (S5)	345	57.97 (20)	8.70 (3)	0.00 (0)	2.90 (1)	2.90 (1)	20.29 (7)
Talamban (S6)	311	51.45 (16)	3.22 (1)	0.00 (0)	6.43 (2)	0.00 (0)	25.72 (8)

^a^n = total number of individuals

^b^number of positive pools out of 24 pools per site

K-W test results (Table 5) showed that seasonal MIRs of CHIKV in *Ae. albopictus* differed (K-W H-test, *H* = 14.814, *df* = 1, *P* < 0.0001), with the mean MIR ranks at 30.69 for the DS and at 51.35 for the WS, but did not differ (K-W H-test, *H* = 0.007, *df* = 1, *P* = 0.932) in the two-site categories by altitude. The mean MIR ranks of CHIKV MIR were 42.27 for highland and 42.73 for lowland. Residents in highland sites stored water in artificial containers for domestic use that served as potential breeding sites for *Aedes* owing to the lack of installed water pipelines by the local government.

**Table 5:** Kruskal-Wallis statistical test results for the minimum infection rates (MIR) of chikungunya (CHIKV), dengue (DENV), and Zika (ZIKV) in *Ae. albopictus* when grouped by season (dry and wet) and by two-site categories by altitude (highland and lowland) in Cebu city, Philippines

Category	Kruskal-Wallis H	df	*P*-value
By season			
CHIKV	14.814	1	0.000*
DENV-1	0.119	1	0.730
DENV-2	4.097	1	0.043*
DENV-3	2.894	1	0.093
DENV-4	11.928	1	0.001*
ZIKV	1.377	1	0.241
By altitude			
CHIKV	0.007	1	0.932
DENV-1	1.960	1	0.162
DENV-2	0.341	1	0.559
DENV-3	0.018	1	0.892
DENV-4	0.108	1	0.743
ZIKV	0.000	1	0.993

*Statistically significant

### MIRs of DENVs in *Ae. albopictus* by season and altitude

During the WS (Table 4; Additional file 2: Dataset 1), only DENV-1 (MIR range: 0–3.21) and DENV-3 (0–12.78) were detected in the highland sites, while DENV-1 (3.22–8.70), DENV-3 (2.90–6.43), and DENV-4 (0–2.90) were detected in the lowland sites. Results suggest that during the WS, *Ae. albopictus* in highland sites might have harbored more DENV-3, while that of the lowland might have harbored more DENV-1. Also, the absence of DENV-2 in *Ae. albopictus* in both two-site categories in the WS suggests that *Ae. aegypti*, the primary dengue mosquito vector, might have maintained DENV-2. During the DS (Table 4), *Ae. albopictus* in both two-site categories had the four DENV serotypes. Results suggest that *Ae. albopictus* played an important role in DENV maintenance during the DS when breeding sites were scarce.

K-W test results (Table 5) revealed that seasonal MIRs of DENV-2 (K-W H-test, *H* = 4.097, *df* = 1, *P* = 0.043) and DENV-4 (K-W H-test, *H* = 11.928, *df* = 1, *P* = 0.001) differed, however, all DENV serotypes when grouped in two-site categories by altitude did not differ (K-W H-test, *P* > 0.05). Results suggest that *Ae. albopictus* circulated all DENV serotypes in both highland and lowland sites, and their residents were exposed to risks of contracting dengue. Moreover, the seasonality of DENV-2 and DENV-4 in *Ae. albopictus* across sites may suggest that local weather might have strongly influenced their viral replications.

### MIRs of DENVs in *Ae. albopictus* and dengue cases in Cebu city

While the combined MIRs of all DENV serotypes in *Ae. albopictus* were high during the DS (March to May 2021 and 2022) and decreased during the start of the WS (July 2021 and June 2022), dengue cases in Cebu city (Additional file 3: Table S2) gradually increased from March 2021 until June 2022 (Fig. 4). Results showed a low, negative, and insignificant correlation (Pearson’s correlation coefficient, *r*_(14)_ = -0.131, *P* = 0.654), implying a weak association between the MIRs of DENVs in *Ae. albopictus* and reported dengue cases. Results suggest that the local *Ae. albopictus* maintained DENVs more frequently during the DS, and transmitted them to humans during the WS.

### MIRs of ZIKV in *Ae. albopictus* by season and altitude

During the DS, the highest MIR (36.81) of ZIKV in *Ae. albopictus* was recorded in Babag 1 highland site, which was also the highest MIR in the entire sampling period (Table 4; Additional file 2: Dataset 1). The lowest MIR (17.75) of ZIKV was recorded in Babag 2, which was also the lowest MIR in the entire study period. During the WS, the highland Taptap site had the highest MIR (31.95) of ZIKV, while that of Babag 2 was lowest (17.91).

The seasonal mean MIRs of ZIKV in *Ae. albopictus* did not differ (K-W H-test, *H* = 1.377, *df* = 1, *P* = 0.241) with 45.14 and 38.99 for the WS and the DS, respectively. Likewise, the mean MIRs of ZIKV were similar (K-W H-test, *H* = 0.000, *df* = 1, *P* = 0.993) between the two-site categories by altitude (Table 5).

### Description of the seasonal weather data

Both the seasonal mean monthly RH (DS 2021 = 76.33%; WS 2021 = 78.50%; DS 2022 = 79%; WS 2022 = 77%) and mean monthly temperatures (DS 2021 = 28.78 °C; WS 2021 = 28.80 °C; DS 2022 = 28.42 °C; WS 2022 = 28.21 °C) were comparable in both WS and DS of 2021 and 2022 sampling years (Table 6). By Student’s t-test, the seasonal monthly RH (t-test, *t*_(11)_ = 2.201, *P* = 0.687) and temperatures (t-test, *t*_(9)_ = 2.262, *P* = 0.862) did not differ. Likewise, the seasonal mean monthly total RF did not differ (t-test, *t*_(6)_ = 2.447, *P* = 0.739) in the WS and DS of 2021 and 2022. Expectedly, the mean monthly total RF was higher in the WS of 2021 (162.40 mm) than that of the DS of the same year (87.60 mm), however, the mean monthly total RF in the DS (2022) (180.13 mm) was higher than that (79.80 mm) of the WS (2022) that apparently could be attributed to climate change.

**Table 6:** Weather data (mean monthly relative humidity [RH] and temperature, and monthly total rainfall [RF]) in Cebu city, Philippines during the dry (March - May 2021; March– May 2022) and wet (June– November 2021; February and June 2022) seasons

Season	Month	RH (%)	RF (mm)	Temperature (ºC)
Dry Season 2021	March	77.00	90.20	28.35
	April	74.00	52.00	28.60
	May	78.00	120.60	29.40
Mean		76.33	87.60	28.78
Wet Season 2021	June	80.00	226.20	28.75
	July	75.00	130.50	29.30
	August	78.00	143.60	28.96
	September	80.00	124.60	28.60
	October	79.00	203.00	28.44
	November	79.00	146.50	28.76
Mean		78.50	162.40	28.80
Dry Season 2022	March	79.00	102.40	28.31
	April	80.00	368.20	27.84
	May	78.00	69.80	29.12
Mean		79.00	180.13	28.42
Wet Season 2022	February	79.00	60.10	27.80
	June	75.00	179.30	28.62
Mean		77.00	119.70	28.21

### Relationship between the MIRs of CHIKV in *Ae. albopictus* and the weather

**Fig. 2:**
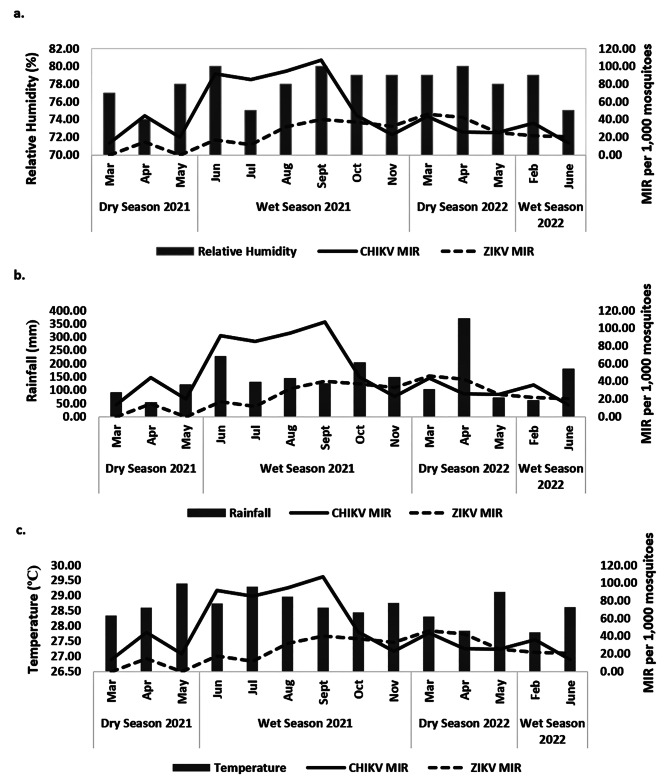
Monthly CHIKV and ZIKV MIR in *Ae. albopictus* with monthly mean relative humidity (%)(**a**), monthly total rainfall (mm)(**b**), and monthly mean temperature (°C)(**c**) in Cebu city, Philippines during the dry (March - May 2021; March– May 2022) and wet (June– November 2021; February and June 2022) seasons. CHIKV, chikungunya; ZIKV, Zika; MIR, minimum infection rate

Figure 2 shows the monthly CHIKV’s MIRs plotted against monthly readings of RH, rainfall, and temperature in Cebu city. CHIKV’s MIRs in *Ae. albopictus* differed (K-W H-test, *H* = 8.537, *df* = 2, *P* = 0.014; Table 7) between rainfall categories (Table 3). The highest mean MIR (52.38) occurred in the moderate RF category (120.1–150.0 mm), followed by high RF category (> 150.0 mm) with mean MIR of 40.42, and lowest at the low RF category (< 120.0 mm) with mean MIR of 34.28. Spearman’s correlation test results revealed a weak positive but insignificant correlation (Spearman’s correlation coefficient, *rs* = 0.16, *P* = 0.573) between the MIR of CHIKV and RF, implying that RF did not positively affect CHIKV. Moreover, CHIKV’s MIRs in *Ae. albopictus* did not differ (K-W H-test, *H* = 3.560, *df* = 2, *P* = 0.169) (Table 7) as influenced by temperature (Table 3) with mean MIRs of 32.04, 45.88, and 39.33 for low (27 °C), moderate (28 °C), and high (29 °C) temperature categories, respectively. Likewise, CHIKV’s MIRs did not differ (K-W H-test, *H* = 4.489, *df* = 2, *P* = 0.106; Table 7) as influenced by RH with mean MIRs of 42.19 (low), 34.19 (moderate), and 47.36 (high) RH categories.

**Table 7:** Kruskal-Wallis statistical test results between the minimum infection rates (MIRs) of chikungunya (CHIKV), dengue (DENV), and Zika (ZIKV) viruses in *Ae. albopictus* and weather data (mean monthly relative humidity [RH] and temperature, and monthly total rainfall [RF]) in Cebu city during the dry (March - May 2021; March– May 2022) and wet (June– November 2021; February and June 2022) seasons

Category	Kruskal-Wallis H	df	*P*-value
By RH			
CHIKV	4.489	2	0.106
DENV-1	0.885	2	0.244
DENV-2	3.073	2	0.215
DENV-3	4.087	2	0.130
DENV-4	2.622	2	0.270
ZIKV	18.106	2	0.000*
By RF			
CHIKV	8.537	2	0.014*
DENV-1	1.422	2	0.491
DENV-2	5.531	2	0.063
DENV-3	0.382	2	0.826
DENV-4	1.503	2	0.472
ZIKV	1.630	2	0.443
By temperature			
CHIKV	3.560	2	0.169
DENV-1	3.426	2	0.180
DENV-2	1.707	2	0.426
DENV-3	0.279	2	0.870
DENV-4	4.815	2	0.090
ZIKV	7.067	2	0.029*

*Statistically significant

### Relationship between the MIRs of ZIKV in *Ae. albopictus* and the weather

Figure 2 shows the monthly ZIKV’s MIR plotted against monthly readings of RH, rainfall, and temperature in Cebu city. ZIKV’s MIR in *Ae. albopictus* differed between categorized ranges (Table 3) for RH (K-W H-test, *H* = 18.106, *df* = 2, *P* < 0.0001) (Table 7) and temperatures (K-W H-test, *H* = 7.067, *df* = 2, *P* = 0.029) (Table 7) but not for RF (K-W H-test, *H* = 1.630, *df* = 2, *P* = 0.443) (Table 7). ZIKV’s MIR was highest in the high RH category (79–80%) with a mean MIR of 53.42, followed by the low monthly RH category (74–76%) with a mean MIR of 34.22, and lowest at moderate monthly RH (77–78%) with a mean MIR of 29.60. A significant positive Spearman’s correlation (Spearman’s correlation coefficient, *rs* = 0.65, *P* = 0.012) between the MIR of ZIKV and RH was observed. On the other hand, ZIKV’s MIR was highest in the low temperature category (27 °C) with a mean MIR of 52.58, followed by moderate (28 °C), and high (29 °C) categories with mean MIRs of 44.27 and 30.47, respectively. A negative and insignificant correlation (Spearman’s correlation coefficient, *rs* = -0.44, *P* = 0.117) was observed between the MIR of ZIKV and temperature.

### Relationship between the MIRs of DENVs in *Ae. albopictus* and weather

**Fig. 3:**
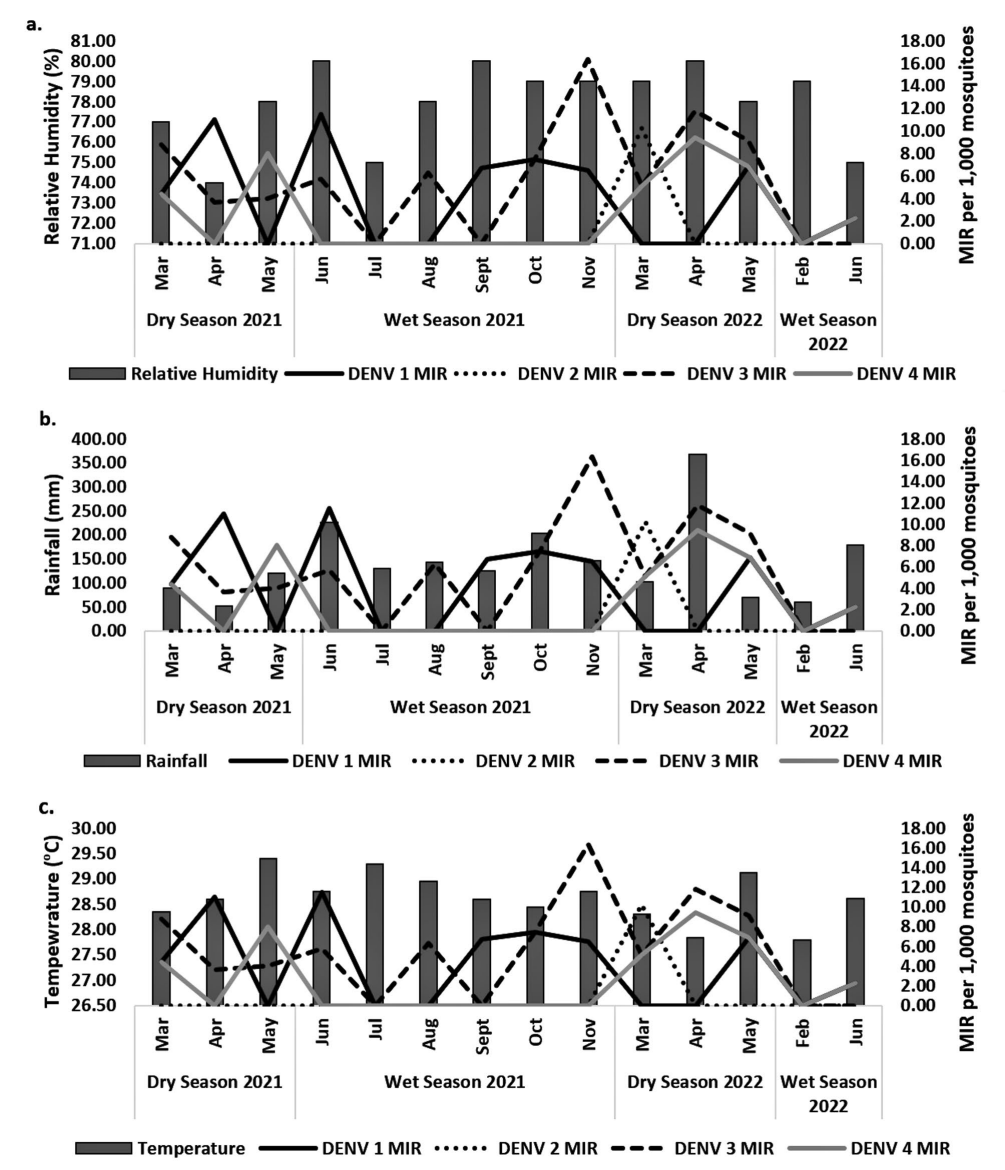
Monthly DENV serotypes (1–4) MIR in *Ae. albopictus* with monthly mean relative humidity (%)(**a**), monthly total rainfall (mm)(**b**), and monthly mean temperature (°C)(**c**) in Cebu city, Philippines during the dry (March - May 2021; March– May 2022) and wet (June– November 2021; February and June 2022) seasons. DENV, Dengue; MIR, minimum infection rate

Figure 3 shows the monthly MIRs of DENVs plotted against monthly readings of RH, rainfall, and temperature in Cebu city. K-W test results showed no differences (K-W H-test, *P* > 0.05) (Table 7) in all DENV serotypes across RH, rainfall, and temperature.

## Discussion

This study is the first report in detecting the impacts of Philippine weather (i.e., RH, RF, and temperature) and seasonal (i.e., WS and DS) conditions on the MIRs of CHIKV, four DENV serotypes, and ZIKV in *Ae. albopictus*, the secondary dengue mosquito vector in the country, collected from selected highlands and lowlands in Cebu city, Philippines during the WS and DS (2021–2022).

### CHIKV in *Ae. albopictus*

First, the seasonal MIRs of CHIKV in *Ae. albopictus* differed but not in two-site categories by altitude (Table 5). The former had higher MIR in the WS (2021–2022), suggesting higher CHIKV prevalence during the WS as supported by the significant difference (Table 7) of CHIKV’s MIRs in *Ae. albopictus* as influenced by RF. Results were consistent with reported positive effect of RF on CHIKV transmission [20, 49–51]. However, similar MIRs of CHIKV in *Ae. albopictus* in both Cebu city’s highland and lowland sites (Table 5) indicate that CHIKV naturally circulated in both two-site categories by altitude. Hence, residents were exposed to risks of contracting CHIKV as evidenced by reported cases described above. Results were consistent in Albanian infestation by CHIKV-infected *Ae. albopictus* at high altitudes [52].

Second, a significant difference of CHIKV’s MIR in *Ae. albopictus* was observed in RF with a weak positive yet insignificant correlation (Spearman’s correlation coefficient, *rs =* 0.16, *P* = 0.573), but not with the RH and temperature (Table 7). The mean monthly total RF (180.13 mm) during the DS (2022) was higher than that (162.4 mm) of the WS (2022), and huge difference of mean monthly total RF between the DS of 2021 (87.6 mm) and 2022 (180.13 mm) (Table 6). Increased precipitation supports the maintenance of breeding sites contributing to mosquitoes’ survival [53]. Currently, the correlation between CHIKV’s prevalence in *Ae. albopictus* and RF is consistent with the reports in Thailand [49, 51] and Kenya [50]. CHIKV is maintained throughout all seasons in Kenya but the abundance of CHIKV-infected mosquitoes is more evident during increased RF (~ 250 mm) [50]. Heavy RF with a three-week interval predicts the onset of CHIKV outbreaks [20]. Although, this study did not observe significant relationship between CHIKV’s MIR in *Ae. albopictus* and mean monthly temperature nearest to the study sites, Mercier et al. [52] reported that *Ae. albopictus* only transmits CHIKV at 28 °C.

### DENVs in *Ae. albopictus*

Currently, *Ae. albopictus* carried all four DENV serotypes (Table 4), like *Ae. aegypti*, consistent with other studies [54–58], although its odds of having DENV-infected saliva is lower than that of *Ae. aegypti* [58]. Interestingly, seasonal MIRs of DENV-2 and DENV-4 in *Ae. albopictus* differed (Table 5), suggesting a specific vector-virus interaction. However, the lack of MIR differences of the four DENV serotypes in *Ae. albopictus* from two-site categories by altitude (Table 5), implies their expanded distribution to highlands as affected by the changing climate [31]. Among the highland sites, Taptap had the highest altitude (719 m ASL), where householders stored water for domestic use in artificial containers [34, 35]. This practice increased the breeding sites in highlands, and may amplify the risks of contracting mosquito-borne diseases. The presence of *Ae. albopictus* in both Cebu city’s lowland (urban) and highlight (rural) sites elucidates their ability to spread these arboviruses [37]. With expanded distribution of *Ae. albopictus* to highlands [59], current vector control programs should be extended there as well.

Currently, the MIRs of DENV-1 and DENV-3 in *Ae. albopictus* were similar with those of *Ae. aegypti* [60], whereas those of DENV-2 and DENV-4 were different. In Brazil, DENV-1 and DENV-3 are most common in *Aedes* and in humans [61]. In Singapore, DENV-1 is most common in both *Ae. aegypti* and *Ae. albopictus* [62]. These studies corroborate with the higher MIRs of DENV-1 and DENV-3 in *Ae. albopictus* from lowland and highland, respectively (Table 4).

**Fig. 4:**
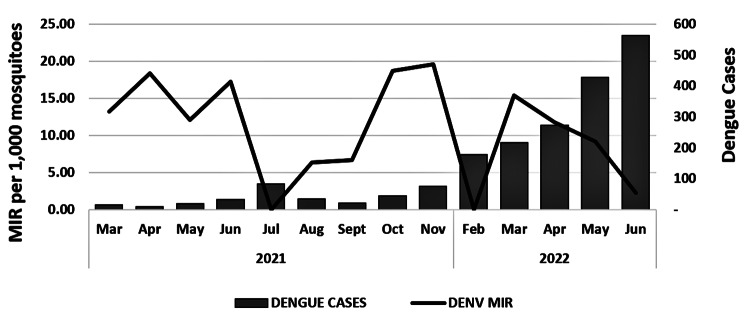
MIRs of combined DENV serotypes (1–4) in *Ae. albopictus* plotted against the reported dengue cases in Cebu city during the wet (June-November 2021; February and June 2022) and dry (March-May 2021 and 2022) seasons. MIR, minimum infection rate; DENV, Dengue

Moreover, *Ae. albopictus* showed higher MIRs of DENVs (mean = 13.33) during the DS, the inter-epidemic period, than during the WS (mean MIR = 8.85) (Table 4), suggesting the silent DENVs’ circulation in mosquitoes on areas with low dengue cases in the DS [63]. In addition, DENV-2 was only detected during the DS regardless of altitude. Hence, *Ae. albopictus* is a good sentinel species in monitoring DENV prevalence during inter-epidemic period [37]. A low negative insignificant correlation (Pearson’s correlation coefficient, *r*_(14)_ = -0.131, *P* = 0.654) between the MIRs of DENV-infected *Ae. albopictus* and dengue cases (Fig. 4) could be attributed to the fact that this species is the secondary dengue vector in the Philippines.

Furthermore, temperature, RF, and RH did not affect the MIRs in DENV-infected *Ae. albopictus* between seasons and altitudes (Table 5). However, these weather predictors influence dengue occurrences and viral transmission in *Ae. aegypti* [35, 62, 64–68]. Generally, DENVs’ mean extrinsic incubation periods (EIP) in vector mosquitoes are 15 days and 6.5 days at 25 and 30 °C, respectively [69]. DENV-2 decreases its EIP in *Ae. albopictus* as temperature increases, with infection rate and transmission efficiency at 31 °C [70]. Atmospheric temperatures (DS: 27.84 to 29.40 °C; WS: 27.80 to 29.30 °C) in Cebu city within the study period (Fig. 3c) were comparable with those of Xiao et al. [70]; *Ae. albopictus* has high infection rates in the head, salivary glands, and thorax-abdomen. DENV-infected *Ae. albopictus* in both lowland (urban) and highland (rural) sites indicates potentially high DENV transmission in both areas [71].

### ZIKV in *Ae. albopictus*

Current results showed a significant difference (Table 7) of ZIKV’s MIRs in *Ae. albopictus* as influenced by RH with positive significant correlation (Spearman’s correlation coefficient, *rs* = 0.65, *P* = 0.012). Studies [72, 73] reported the correlation between ZIKV detection in *Ae. albopictus* and RH, particularly in its survival, fecundity, and egg-hatching. At 47–52% RH, this species’ fecundity and longevity are better than other *Aedes* species [74]. *Ae. albopictus’* longevity is relatively better at higher RH (85%) than lower RH (35%) [73].

Second, ZIKV’s MIRs in *Ae. albopictus* differed as influenced by temperature was significant (Table 7), although with negative insignificant correlation (Spearman’s correlation coefficient, *rs* = -0.44, *P* = 0.117). ZIKV’s MIR was higher at 27 ºC, consistent with Mordecai et al. [17], who reported that ZIKV transmission occurs from 22.7 to 34.7 °C. The optimum is at 30.6 °C (range: 22.9 to 38.4 °C) for ZIKV vector competence in *Ae. aegypti* [75]. At constant 23 °C, adult *Ae. albopictus* carries higher ZIKV’s RNA load [75]. ZIKV transmission rate by *Ae. albopictus* in Europe is influenced also by the viral load and temperature [76]. These studies [17, 74–76] suggest that temperature drives ZIKV transmission by *Ae. aegypti* and *Ae. albopictus*; beyond the maximum thermal range, ZIKV transmission may decline. This might be attributed to the current study’s negative correlation between MIR of ZIKV in *Ae. albopictus* and temperature.

Third, the mean MIR of ZIKV in *Ae. albopictus* did not differ by season and by two-site categories (Table 4). The latter result was consistent between *Ae. albopictus* populations at highest and lowest altitudes in Albania [52]. Current results may suggest that *Ae. albopictus* transmitted ZIKV all throughout the year and in both lowland and highland sites in Cebu city. Moreover, results imply a whole-year round implementation of the DOH enhanced *4 S* strategy in this climate change as in related studies [34, 35].

## Conclusion

In conclusion, the MIRs of CHIKV in *Ae. albopictus* were affected by RF, whereas those of ZIKV were affected by RH and temperature. DENVs in *Ae. albopictus* varied seasonally; DENV-2 and DENV-4 differed seasonally across selected highland and lowland study sites in Cebu city. Seasonal temperature, RF, and RH did not influence the MIRs of DENVs in *Ae. albopictus* across the two-site categories by altitude. One limitation of this study was the use of PAG-ASA weather data nearest to the study sites but were not measured on-site. However, this study aimed to explore the relationships between the MIRs of the arboviruses in the local study sites with the weather data, hence, an encompassing weather data of Cebu was assumed sufficient to demonstrate such relationships.

This study recommends for an all-year round implementation of the Philippine DOH’s enhanced *4 S* strategy in this climate change because of the lack of the seasonal impact on ZIKV, DENV-1 and DENV-3 in *Ae. albopictus*; similar as in our previous studies [34, 35]. Water pipelines [34, 35] and vector control services are highly recommended in the highlands and not just in the lowlands to minimize the practice in storing water in containers that serve as potential breeding sites of dengue mosquitoes as mentioned in the above results. The MIR data of these three arboviruses in *Ae. albopictus* will be useful in future arboviral modeling at which weather conditions can potentially be used as indicators in predicting possible arboviral outbreaks. Thus, findings of this study are very relevant not just in the Philippines but also in the tropics and subtropics.

### Electronic supplementary material

Below is the link to the electronic supplementary material.

Additional file 1: Table S1. Primer sequences for arboviral detection.

Additional file 2: Dataset S1. Minimum infection rates (MIRs) for Chikungunya (CHIKV), Dengue (DENV) and its serotypes, and Zika (ZIKV) from adult *Ae. albopictus* pools collected in Cebu city highland (S1-S3) and lowland (S4-S6) sites in dry (March-May 2021 and March-May 2022) and wet seasons (June-November 2021; February and June 2022).

Additional file 3: Table S2. Dengue cases in Cebu city, Philippines in dry (March-May 2021 and March-May 2022) and wet seasons (June-November 2021; February and June 2022).

## Data Availability

The meteorological data and dengue cases supporting the conclusions of this article are included within the article and its additional files. They are also available from Philippine Atmospheric, Geophysical and Astronomical Services Administration (PAGASA) and Department of Health, respectively, though only accessible upon request through the Philippine Freedom of Information (foi.gov.ph).

## Abbreviations

CHIKV: Chikungunya
DENV: Dengue
ZIKV: Zika
H: Highland
L: Lowland
WS: Wet season
DS: Dry season
MIR: Minimum infection rate
RF: Rainfall
RH: Relative humidity
CFR: Case fatality rate
m ASL: Meters above sea level
O/L: Ovicidal/larvicidal
USC-MRL: University of San Carlos Mosquito Research Laboratory
DW: Distilled water
RT-PCR: Reverse transcriptase polymerase chain reaction
dNTP: Deoxynucleotide triphosphate
RFW: RNAse-free water
NFW: Nuclease-free water
PGC: Philippine Genome Center
PAG-ASA: Philippine Atmospheric Geophysical and Astronomical Services Administration
K-W: Kruskal-Wallis
