# Supplementary material for: Detecting the impacts of humidity, rainfall, temperature, and season on chikungunya, dengue and Zika viruses in Aedes albopictus mosquitoes from selected sites in Cebu city, Philippines
Source: Virol J. 2024 Feb 15;21:42. doi: 10.1186/s12985-024-02310-4 (PMC10870450; doi:10.1186/s12985-024-02310-4)
Supplement: Supplementary file 1 — Additional file 1: Table S1. Primer sequences for arboviral detection. [file 12985_2024_2310_MOESM1_ESM.pdf]

Additional file 1: Table S1. Primer sequences for arboviral detection.

Primer	Sequence
CHIKV	
6K/E1 OA	5’-AGTCAACAGACCGGGCTACA-3’
6K/E1 OB	5’-CGCTCAATTGCGTATTTTCA-3’
6K/E1 IA	5’-GCCCAATGGTACTGGAGATG-3’
6K/E1 IB	5’-GTGTCGCAGAAGCAGTAGGC-3’
DENV	
D1	5’-TCAATATGCTGAAACGCGCGAGAAACCG-3’
D2	5’-TTGCACCAACAGTCAATGTCTTCAGGTTC-3’
TS1	5’-CGTCTCAGTGATCCGGGGG-3’
TS2	5’-CGCCACAAGGGCCATGAACAG-3’
TS3	5’-TAACATCATCATGAGACAGAGC-3’
TS4	5’CTCTGTTGTCTTAAACAAGAGA-3’
ZIKV	
ZIKV F	5’CCTTGGATTCTTGAACGAGGA-3’
ZIKV R	5’-AGAGCTTCATTCTCCAGATCAA-3’
