# Supplementary material for: Detecting the impacts of humidity, rainfall, temperature, and season on chikungunya, dengue and Zika viruses in Aedes albopictus mosquitoes from selected sites in Cebu city, Philippines
Source: Virol J. 2024 Feb 15;21:42. doi: 10.1186/s12985-024-02310-4 (PMC10870450; doi:10.1186/s12985-024-02310-4)
Supplement: Supplementary file 3 — Additional file 3: Table S2. Dengue cases in Cebu city, Philippines in dry (March-May 2021 and March-May 2022) and wet seasons (June-November 2021; February and June 2022). [file 12985_2024_2310_MOESM3_ESM.pdf]

Additional file 32: Table S2. Dengue cases in Cebu city, Philippines in dry (March-May 2021 and March-May 2022) and wet seasons (June-November 2021; February and June 2022).

Season	Month	Dengue cases
Dry Season 2021	March	15
	April	10
	May	19
Wet Season 2021	June	33
	July	82
	August	35
	September	20
	October	45
	November	76
Dry Season 2022	March	217
	April	272
	May	427
Wet Season 2022	February	177
	June	562
